# 全脑放疗时间对*EGFR*突变非小细胞肺癌脑转移患者生存的影响

**DOI:** 10.3779/j.issn.1009-3419.2016.08.03

**Published:** 2016-08-20

**Authors:** 桂梅 刘, 新勇 张, 翠孟 田, 广荣 夏, 平 刘, 权 张, 曦 李, 卉 张, 娜 秦, 敬慧 王, 树才 张

**Affiliations:** 1 101149 北京，首都医科大学附属北京胸科医院，北京市结核病胸部肿瘤研究所放疗科 Department of Radiotherapy, Beijing Tuberculosis and Thoracic Tumor Research Institute, Beijing 101149, China; 2 101149 北京，首都医科大学附属北京胸科医院, 肿瘤内科 Department of Medical Oncology, Beijing Chest Hospital, Capital Medicine University, Beijing Tuberculosis and Thoracic Tumor Research Institute, Beijing 101149, China

**Keywords:** 全脑放疗, 酪氨酸激酶抑制剂, 表皮生长因子受体突变, 肺肿瘤, 脑转移, Whole brain radiotherapy, Tyrosine kinase inhibitors, Epidermal growth factor receptor mutation, Lung neoplasms, Brain metastasis

## Abstract

**背景与目的:**

全脑放疗（whole brain radiotherapy, WBRT）在表皮生长因子受体（epidermal growth factor receptor, *EGFR*）突变的非小细胞肺癌（non-small cell lung cancer, NSCLC）脑转移患者治疗中何时应用尚无高级别的循证医学证据。本研究旨在探讨WBRT的参与时间对携有*EGFR*突变的NSCLC脑转移患者生存的影响。

**方法:**

2009年8月-2015年5月在我院确诊的*EGFR*突变伴脑转移的晚期NSCLC共78例患者，均接受WBRT及EGFR酪氨酸激酶抑制剂（EGFR tyrosine kinase inhibitors, EGFR-TKIs）治疗的48例初治患者进入临床分析，采用*Cox*比例风险模型分析患者颅内无进展生存期（progression-free survival, PFS）及总生存期（overall survival, OS）的影响因素。

**结果:**

全组患者颅内客观缓解率（objective response rate, ORR）为81.3%，颅内疾病控制率（disease control rate, DCR）为93.8%，中位颅内PFS为10个月，中位OS为18个月。颅内PFS的多因素分析显示，美国东部肿瘤协作组评分（Eastern Cooperative Oncology Group performance status, ECOG PS）0分-1分（HR=30.436, 95%CI: 4.721-196.211, *P* < 0.001）及早期WBRT患者（HR=3.663, 95%CI: 1.657-8.098, *P*=0.001）的颅内PFS更佳。OS的多因素分析显示，ECOG PS 0分-1分（HR=57.607, 95%CI: 6.135-540.953, *P* < 0.001）、早期WBRT（HR=2.757, 95%CI: 1.140-6.669, *P*=0.024）及立体定向放射外科（stereotaxic radio surgery, SRS）的应用（HR=5.964, 95%CI: 1.895-18.767, *P*=0.002）是患者OS的独立预后因素。

**结论:**

早期WBRT联合TKIs治疗可改善*EGFR*突变的NSCLC脑转移患者的预后，尚有待大样本的前瞻性临床试验验证。

## Abstract (en)

**Background and objective:**

There is no high-level evidence for the time of whole brain radiotherapy (WBRT) for patients with epidermal growth factor receptor (*EGFR*)-mutated non-small cell lung cancer (NSCLC) and brain metastases.The aim of this study is to assess the appropriate timing of WBRT for patients with *EGFR*-mutated NSCLC and brain metastases (BM).

**Methods:**

There were 78 patients diagnosed with *EGFR*-mutated NSCLC and BM in Beijing Chest Hospital between August 2009 and May 2015.48 untreated patients who received both WBRT and EGFR-tyrosine kinase inhibitors (TKIs) therapy.Prognostic factors of intracranial progression-free survival (PFS) and overall survival (OS) were identified by *Cox* proportional hazards modeling.

**Results:**

Intracranial objective response rate was 81.3% and disease control rate was 93.8%.Median intracranial PFS was 10 months.Median OS was 18 months.Multivariate analysis of intracranial PFS revealed that Eastern Cooperative Oncology Group (ECOG) performance status (PS) 0-1 (HR=30.436, 95%CI:4.721-196.211, *P* < 0.001) and early WBRT (HR=3.663, 95%CI:1.657-8.098, *P*=0.001) had a better intracranial PFS.Multivariate analysis of OS revealed that PS 0-1 (HR=57.607, 95%CI:6.135-540.953, *P* < 0.001), early WBRT (HR=2.757, 95%CI:1.140-6.669, *P*=0.024), and stereotactic radiosurgery (HR=5.964, 95%CI:1.895-18.767, *P*=0.002) were independent prognostic factors of OS.

**Conclusion:**

Early WBRT combined with EGFR-TKIs can improve outcomes of patients with *EGFR*-mutated NSCLC and BM, but it needs to be confirmed by large-sample-size and multicenter prospective clinical trials.

全球范围内肺癌的发病率和死亡率均居恶性肿瘤首位^[1]^, 其中非小细胞肺癌(non-small cell lung cancer, NSCLC)占75%-80%, 约25%-38%NSCLC患者在整个病程中发生脑转移事件^[2]^。NSCLC脑转移自然生存期仅4周-6周^[3]^, 全脑放疗(whole brain radiotherapy, WBRT)是标准治疗方案, 中位总生存时间(overall survival, OS)延长到4个月-6个月^[4]^。随着外科手术和立体定向放射外科(stereotactic radiosurgery, SRS)的介入提高OS达到10个月^[5]^。多项临床试验^[6, 7]^证实, 表皮生长因子受体(epidermal growth factor receptor, EGFR)酪氨酸激酶抑制剂(tyrosine kinase inhibitors, TKIs)能明显延长*EGFR*突变的晚期NSCLC的疾病无进展时间(progression-free survival, PFS), 已成为这类患者一线治疗的标准方案。携有*EGFR*敏感突变的晚期NSCLC脑转移患者除了传统的放疗、化疗, 增加了EGFR-TKIs靶向治疗机会。但是, 脑部放疗如何选择和安排更有益于患者, 目前尚无高级别的循证医学证据。本项研究对首都医科大学附属北京胸科医院*EGFR*突变的NSCLC脑转移患者的治疗及生存情况进行回顾分析, 探讨WBRT的应用时间对此类患者生存的影响。

## 1 资料与方法

### 1.1 临床资料

2009年8月-2015年5月在首都医科大学附属北京胸科医院经病理学诊断为*EGFR*敏感突变伴脑转移的NSCLC患者78例, 剔除EGFR-TKIs治疗后出现的脑转移患者14例、脑转移诊断后单独WBRT 2例、单独化疗5例、放疗联合化疗3例、化疗联合TKIs 3例、未治疗3例, 均接受WBRT和EGFR-TKIs治疗的48例初治患者进入临床分析, 患者的一般情况见表 1。48例患者中, 43例初诊即为肺癌伴脑转移, 5例为初诊肺癌接受根治手术后出现脑转移。患者伴有中枢神经系统症状的常见表现有头痛、头晕、恶心、呕吐、言语不清或肢体活动障碍等。

**1:** 患者的一般资料 Basic clinical characteristicsof patients (*n*=48)

Characteristics	*n*
Age (year)	
Median	57
Range	30-83
≤57	26 (54.2%)
> 57	22 (45.8%)
Gender	
Male	16 (33.3%)
Female	32 (66.7%)
Smoking	
No	35 (72.9%)
Yes	13 (27.1%)
ECOG performance status (PS)	
0-1	46 (95.8%)
2	2 (4.2%)
Histological type	
Adenocarcinoma	46 (95.8%)
Squamous	2 (4.2%)
No.of brain lesions	
< 3	14 (29.2%)
≥3	34 (70.8%)
Initial brain symptom	
Yes	27 (56.3%)
No	21 (43.7%)
No.of extracranial metastases	
No	6 (12.5%)
One site	11 (22.9%)
Multiple sites	31 (64.6%)
*EGFR* mutation	
Exon 19 deletion	25 (52.1%)
Exon 21 L858R	23 (47.9%)
ECOG:Eastern Cooperative Oncology Group; EGFR:epidermal growth factor receptor.	

### 1.2 治疗方法

所有患者治疗前均行血常规、血生化、心电图、浅表淋巴结彩超、腹部彩超、骨扫描、胸部计算机断层扫描(computed tomography, CT)、头颅增强CT或增强磁共振成像(magnetic resonance imaging, MRI)等常规检查。

#### 1.2.1 放疗方法

采用Siemens或Varian加速器, Philips CT模拟定位, 计划系统采用Prowess或Eclipse三维治疗计划系统, WBRT根据CT影像学勾画临床靶区(clinical target volume, CTV), 限定危及器官受量:晶体≤8 Gy, 脑干≤50 Gy, 眼球≤45 Gy, 视神经和视交叉≤50 Gy, 放疗采用6 Mv-10 Mv X射线, WBRT剂量30 Gy/10次; SRS在外院进行, 50%剂量曲线15 Gy-18 Gy。早放疗指患者诊断脑转移后立即进行WBRT并联合TKIs或化疗, 晚放疗指患者诊断脑转移后先行化疗或TKIs治疗, 颅内进展后再进行WBRT。48例患者中早放疗32例(66.7%), 晚放疗16例(33.3%)。11例(22.9%)接受SRS治疗, 其中一线SRS 5例, 补救性SRS治疗6例。

#### 1.2.2 EGFR-TKIs治疗

吉非替尼250 mg, 口服, 1次/日; 厄洛替尼150 mg, 口服, 1次/日; 埃克替尼125 mg, 口服, 3次/日。一线EGFR-TKIs治疗为患者诊断为脑转移后开始应用, 直到疾病进展或死亡或发生不可耐受的不良反应; 二线EGFR-TKIs为患者进行一线化疗或一线化疗联合放疗, 疾病进展进行的EGFR-TKIs治疗。48例患者中, 39例(81.3%)为TKIs一线治疗, 9例(18.7%)为二线治疗。

#### 1.2.3 化疗

48例患者中17例(35.4%)患者接受化疗, 其中一线化疗9例, 二线化疗8例。化疗方案有单药培美曲塞、培美曲塞+顺铂、多西他赛+顺铂、诺维本+顺铂、培美曲塞+卡铂。

### 1.3 疗效及不良反应评价

治疗前均有头颅增强CT或增强MRI对颅内病灶进行评估, 治疗后1个月复查评价疗效, 以后每2-3个月随访1次。按实体瘤疗效评价标准(response evaluation criteria in solid tumors, RECIST)1.1版评价近期疗效, 分为完全缓解(complete response, CR)、部分缓解(partial response, PR)、疾病稳定(stable disease, SD)和疾病进展(progressive disease, PD)。客观缓解率(objective response rate, ORR)以CR+PR计算, 疾病控制率(disease control rate, DCR)以CR+PR+SD计算。不良反应按NCI CTC 3.1标准分为0-4级。

### 1.4 随访资料

颅内疾病无进展时间(progression-free survival, PFS)为治疗开始到颅内病灶进展或死亡的时间, OS为从确诊脑转移至任何原因所致死亡的时间。随访截止时间为2016年5月10日, 存活患者按照删失数据处理。随访方式为门诊随访及电话随访, 随访率为100%。

### 1.5 统计学方法

采用统计软件SPSS 22.0进行数据处理。计数资料采用卡方检验或精确概率法; 采用*Kaplan-Meier*进行生存分析, 组间差异采用*Log-rank*检验, 多因素分析采用*Cox*比例风险模型。*P*值采用双侧检验, *P* < 0.05为差异有统计学意义。

## 2 结果

### 2.1 近期疗效

全组患者均可评价疗效, CR为8例, PR为31例, SD为6例, PD为3例。全组患者颅内ORR为81.3%, 颅内DCR为93.8%。年龄、性别、吸烟状况、PS评分、病理类型、病灶数目、神经系统症状、颅外转移部位、突变类型、WBRT时间、SRS与否、TKIs时间及化疗与否对颅内ORR均无影响(*P* > 0.05)。

### 2.2 颅内中位PFS

全组患者中33例(68.8%)进展, 15例未进展, 中位颅内PFS为10个月(95%CI:8.086-11.914)。对48例患者颅内PFS进行单因素分析, 结果显示, PS 0-1分、无颅外转移及早放疗患者的中位PFS优于PS 2分、有颅外转移及晚放疗患者。将单因素分析中具有统计学意义的变量进行*Cox*多因素分析, 结果显示PS 0-1分(HR=30.436, 95%CI:4.721-196.211, *P* < 0.001;图 1A)与早放疗(HR=3.663, 95%CI:1.657-8.098, *P*=0.001;图 1B)患者的颅内中位PFS明显延长。

**1:**
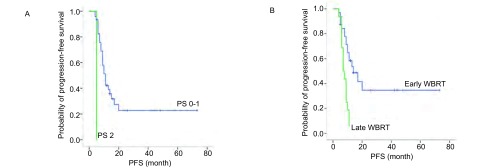
不同PS评分的颅内无进展生存期曲线(A)和不同全脑放疗时间的颅内无进展生存期曲线(B) Intracranial progression-free survival(PFS) according to PS score(A) and time of whole brain radiotherapy(B)

### 2.3 中位OS

全组患者中31例(64.5%)死亡, 17例存活, 中位OS为18个月(95%CI:14.832-21.168)。单因素分析显示, PS评分、神经系统症状、WBRT时间、SRS与否及化疗与否与中位OS相关。差异有统计学意义的单因素进入*Cox*多因素分析, 结果显示PS 0-1(HR=57.607, 95%CI:6.135-540.953, *P* < 0.001;图 2A)、早放疗(HR=2.757, 95%CI:1.140-6.669, *P*=0.024;图 2B)及SRS的应用(HR=5.964, 95%CI:1.895-18.767, *P*=0.002;图 2C)是OS的独立预测因素。全组患者颅内PFS及OS单因素分析结果见表 2。

**2:**
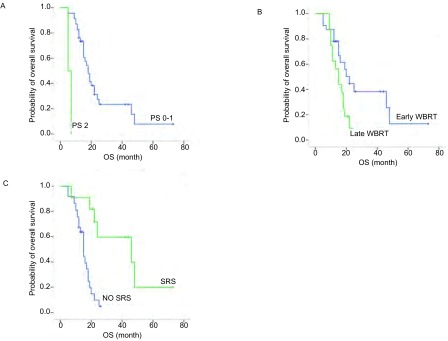
总生存期曲线。A:PS评分; B:全脑放疗时间; C:接受SRS与否。 *Kaplan-Meier* survival curve of overall survival (OS).A:PS score; B:Time of whole brain radiotherapy; C:SRS or not.

**2:** 48例患者颅内无进展中位生存时间及总生存时间的单因素分析 Univariateanalysis of intracranial PFS and OS for 48 patients

Item	*n*	PFS				OS		
		Median (month)	95%CI	*P*		Median (month)	95%CI	*P*
Age (yr)				0.256				0.060
≤57	26	10.0	7.560-12.440			22.0	17.730-26.270	
> 57	22	10.0	7.242-12.758			15.0	13.092-16.908	
Gender				0.267				0.316
Male	16	17.0	10.169-23.831			22.0	17.393-26.607	
Female	32	9.0	7.156-10.844			15.0	11.825-18.175	
Smoking status				0.614				0.571
No	35	10.0	7.516-12.484			18.0	15.647-20.353	
Yes	13	10.0	7.737-12.263			15.0	11.390-18.610	
PS score				< 0.001				< 0.001
0-1	46	11.0	9.147-12.853			18.0	14.877-21.123	
2	2	5.0	-			5.0	-	
Histological type				0.309				0.365
Adenocarcinoma	46	10.0	8.126-11.874			18.0	14.830-21.170	
Squamous	2	5.0	-			7.0	-	
No.of brain lesions								
< 3	14	9.0	6.555-11.445	0.583		18.0	15.926-20.074	0.999
≥3	34	11.0	9.171-12.829			18.0	11.926-24.074	
Intial brain symptom				0.172				0.042
Yes	27	11.0	7.790-14.210			22.0	18.084-25.916	
No	21	9.0	6.037-11.963			15.0	9.778-20.222	
No.of extracranial metastases				0.046				0.191
No	6	-	-			20.0	-	
One site	11	9.0	6.842-11.158			15.0	10.609-19.391	
Multiple sites	31	10.0	7.998-12.002			18.0	13.930-22.070	
*EGFR* mutation				0.433				0.734
*Exon* 19 deletion	25	10.0	8.426-11.574			19.0	17.144-20.856	
*Exon* 21 L858R	23	11.0	9.447-12.553			15.0	11.247-18.753	
Time of radiotherapy				< 0.001				0.008
Early	32	14.0	7.170-20.830			22.0	16.128-27.872	
Late	16	7.0	5.040-8.960			15.0	11.111-18.889	
SRS				0.245				< 0.001
Yes	11	17.0	4.053-29.947			46.0	3.922-88.078	
No	37	10.0	8.326-11.674			15.0	13.019-16.981	
Treatment line of TKIs				0.967				0.127
First line	39	11.0	8.619-13.381			16.0	12.875-19.125	
Second line	9	10.0	8.614-11.386			22.0	6.984-37.016	
Chemotherapy				0.742				0.046
Yes	17	10.0	8.011-11.989			22.0	14.984-29.016	
No	31	11.0	8.351-13.649			16.0	11.675-20.325	
PFS:progression-free survival; OS:overall survival; TKI:tyrosine kinase inhibitor; SRS:stereotactic radiosurgery.								

### 2.4 不良反应

治疗期间最常见的是脱发、头晕、呕吐, 治疗过程中都给予脱水和激素等降低颅内压治疗及对症处理, EGFR-TKIs治疗最常见的不良反应有皮疹、腹泻、恶心、转氨酶升高等, 多为1级或2级, 可以耐受, 无治疗相关的死亡发生。

## 3 讨论

本项研究对我院48例确诊为*EGFR*敏感突变的NSCLC脑转移的患者进行回顾性分析(*EGFR*敏感突变接受TKIs治疗后出现脑转移的患者未纳入本研究), 探讨这些患者治疗转归的影响因素, 尤其是放疗时间对PFS和OS的影响。48例患者均接受了WBRT和EGFR-TKIs治疗。全组的颅内ORR为81.3%, 中位颅内PFS为10个月, 中位OS为18个月, 多因素分析显示, 确诊脑转移后即开始放疗(即早放疗)能明显延长颅内PFS和OS, 优于晚放疗(即脑转移进展后开始放疗)。

传统化疗药物进入脑内受到血脑屏障的阻碍等因素, 药物对脑转移的治疗难以达到理想的效果, 脑转移是肺癌死亡的主要原因之一。而一直以来, 局部治疗如WBRT、SRS等是脑转移治疗的主要方法。随着肺癌分子分型和靶向药物的发展, EGFR-TKIs成为*EGFR*突变患者的一线治疗选择, 包括脑转移的患者。EGFR-TKIs是小分子药物, 可通过血脑屏障进入颅内, 对脑转移也有较好的控制效果。Park等^[8]^报道28例*EGFR*突变肺癌脑转移患者仅接受TKI治疗的颅内PFS为6.6个月, OS为15.9个月, 此研究取得较长生存时间是因为病变进展后又接受了放疗作为补救治疗, 17例脑部进展患者有14例进行放疗(11例SRS、3例WBRT)。因此, 单独采用TKI治疗NSCLC脑转移尚显不足。另外Iuchi等^[9]^也有同类研究发表。

Ma等^[10]^的Ⅱ期研究报告WBRT联合吉非替尼治疗NSCLC脑转移, ORR和DCR分别为81%和95%, OS为13个月。Welsh等^[11]^的Ⅱ期临床研究也显示, WBRT联合厄洛替尼治疗NSCLC脑转移的ORR达到86%, *EGFR*突变患者中位OS达到了19.1个月。Zhou等^[12]^报告在一项埃克替尼剂量递增的Ⅰ期研究中, 埃克替尼联合WBRT的颅内ORR为80%, 颅内PFS达到18.9个月, 中位OS还未达到, 证实WBRT联合TKIs治疗能获得更为明显的颅内疾病控制和生存。本研究患者均为*EGFR*敏感突变患者, 全部接受放疗和EGFR-TKIs治疗, 全组患者颅内ORR为81.3%, 颅内DCR为93.8%, 中位OS为18个月。本文结果与上述研究结果相似。患者接受EGFR-TKIs联合WBRT在颅内PFS优于单独EGFR-TKIs, 并且获得较为理想的生存, 总生存接近几项大的随机临床试验, 如IPASS、OPTIMAL等报告的突变人群的总生存^[6, 7]^。同时, 联合治疗有基础研究的支持, EGFR-TKI与放疗联合对控制肿瘤有协同作用^[13]^。也有研究^[14]^报告厄洛替尼可通过血脑屏障, 提高WBRT的疗效。

对于脑部放疗联合TKIs治疗*EGFR*敏感突变脑转移患者的预后分析的研究少有报告。我们对PFS和OS的影响因素进行*Cox*多因素分析, 结果显示, PS 0-1分和早放疗是PFS的独立预后因素, PS 0-1分、早放疗和SRS的应用是OS的独立影响因素, 接受早放疗患者的颅内PFS和OS均得到明显延长。其他因素, 如颅内转移灶的多少、颅外转移、突变类型及EGFR-TKIs一线或二线使用等, 颅内PFS和OS未见差异, 显示WBRT作为局部治疗的重要手段早期应用协同TKI治疗可提高颅内PFS及OS, 说明早期进行WBRT的重要性。Soon等^[15]^的*meta*分析报告, 先行脑部放疗联合全身治疗较单独TKIs可提高颅内疾病控制和总生存。本文结果与这项*meta*分析的结论一致。

SRS是肿瘤脑转移患者重要的局部治疗方式之一, 与WBRT相比具有更加精准、局部控制率高、对患者认知功能影响小等优点, 并可以与WBRT联合应用。SRS联合WBRT在控制脑转移方面优于单一的治疗手段, 联合治疗可明显提高患者颅内肿瘤控制率^[16]^。一项随机试验^[17]^证实, 1个-3个脑转移灶患者应用SRS+WBRT相比单独应用WBRT能提高OS(6.5个月*vs* 4.9个月, *P*=0.039, 3), 同时SRS可以作为WBRT后复发患者的补救性治疗^[18]^。本组结果显示, SRS参与是OS的独立预后因素, 但并未延长PFS时间。对接受SRS治疗的患者进行分析, 全组48例患者中11例做了SRS治疗, 其中5例为一线应用, 即与WBRT同时进行, 5例中的3例的OS分别为49个月、58个月、81个月, 2例仍存活; 6例SRS为补救性治疗, 即脑转移进展后的治疗。多因素分析显示联合SRS能明显延长OS, 提示对于脑转移患者, 无论何时应用SRS, 患者的OS均获益, 脑转移患者在治疗过程中应联合应用SRS。本文中未显示出SRS对PFS有影响, 原因可能与本组患者仅5例一线应用, 与例数少有关。当前更多的治疗*EGFR*突变患者的一代、二代、三代药物进入临床, 患者的生存期明显延长, 为更好地使患者获得颅内病变控制, 并降低认知功能受损的发生, 是否有条件的建议可先行SRS, WBRT推迟应用值得进一步探索。多项治疗脑转移的临床试验, 如EGFR-TKI与SRS联合、TKI联合WBRT对比WBRT的Ⅲ期研究正在进行中。

综上所述, 早期WBRT治疗联合EGFR-TKIs能明显延长*EGFR*突变NSCLC脑转移患者的颅内PFS和OS, SRS应用有助于改善生存。本研究为回顾性分析, 例数偏少, 存在一定的偏倚, 且脑转移患者的混杂因素较多, 亟需设计良好的前瞻性临床试验探索此类患者的最佳治疗方案。
